# Supplementary material for: Learning compositional sequences with multiple time scales through a hierarchical network of spiking neurons
Source: PLoS Comput Biol. 2021 Mar 25;17(3):e1008866. doi: 10.1371/journal.pcbi.1008866 (PMC8023498; doi:10.1371/journal.pcbi.1008866)
Supplement: S1 Text — We extend the model such that it can learn sequences consisting of more than two motifs, with variable durations. In the main text, each motif has the same duration. This means the supervisor only needs to provide a starting signal to the fast clock, indicating when a motif starts. In general, a motif can be shorter than a fast clock sequence. In that case, the supervisor has to provide a stop signal to the fast clock, indicating when a motif ends. This stop signal activates the penultimate cluster in the fast clock, which activates in turn the ‘silent’ interneurons. The stop signal is 10 ms long and has the same rate as the start signal. We learn example sequences to illustrate this (S6 Fig). Specifically, we learn sequences ABCD and EBCF. Motifs A and B are both 200 ms long. Motif C, D, E and F are respectively 150 ms, 120 ms, 180 ms and 100 ms long. To keep the sequences as general as possible, we also include variable inter-motif intervals. The silent gap between motifs A and B, motifs B and C, and motifs C and D is respectively 70 ms, 50 ms, and 80 ms. The silent gaps in the second sequence between motifs E and B, motifs B and C, and motifs C and F are respectively 70 ms, 50 ms, and 150 ms. We observe that the model is able to learn the two sequences, but the replay of shorter motifs D and F is less accurate. The parameters used in this simulation are the same as in other simulations, with an increased network size for the interneuron networks, and read-out network. (PDF) [file pcbi.1008866.s001.pdf]

S1 Text Extending the model to more and variable motif lengths. We extend the model such that it can learn sequences consisting of more than two motifs, with variable durations. In the main text, each motif has the same duration. This means the supervisor only needs to provide a starting signal to the fast clock, indicating when a motif starts. In general, a motif can be shorter than a fast clock sequence. In that case, the supervisor has to provide a stop signal to the fast clock, indicating when a motif ends. This stop signal activates the penultimate cluster in the fast clock, which activates in turn the 'silent' interneurons. The stop signal is 10 ms long and has the same rate as the start signal. We learn example sequences to illustrate this (S6 Fig). Specifically, we learn sequences $ABCD$ and $EBCF$. Motifs A and B are both 200 ms long. Motif C, D, E and F are respectively 150 ms, 120 ms, 180 ms and 100 ms long. To keep the sequences as general as possible, we also include variable inter-motif intervals. The silent gap between motifs A and B, motifs B and C, and motifs C and D is respectively 70 ms, 50 ms, and 80 ms. The silent gaps in the second sequence between motifs E and B, motifs B and C, and motifs C and F are respectively 70 ms, 50 ms, and 150 ms. We observe that the model is able to learn the two sequences, but the replay of shorter motifs D and F is less accurate. The parameters used in this simulation are the same as in other simulations, with an increased network size for the interneuron networks, and read-out network.
