# Supplementary figures and images for: Learning compositional sequences with multiple time scales through a hierarchical network of spiking neurons
Source: PLoS Comput Biol. 2021 Mar 25;17(3):e1008866. doi: 10.1371/journal.pcbi.1008866 (PMC8023498; doi:10.1371/journal.pcbi.1008866)

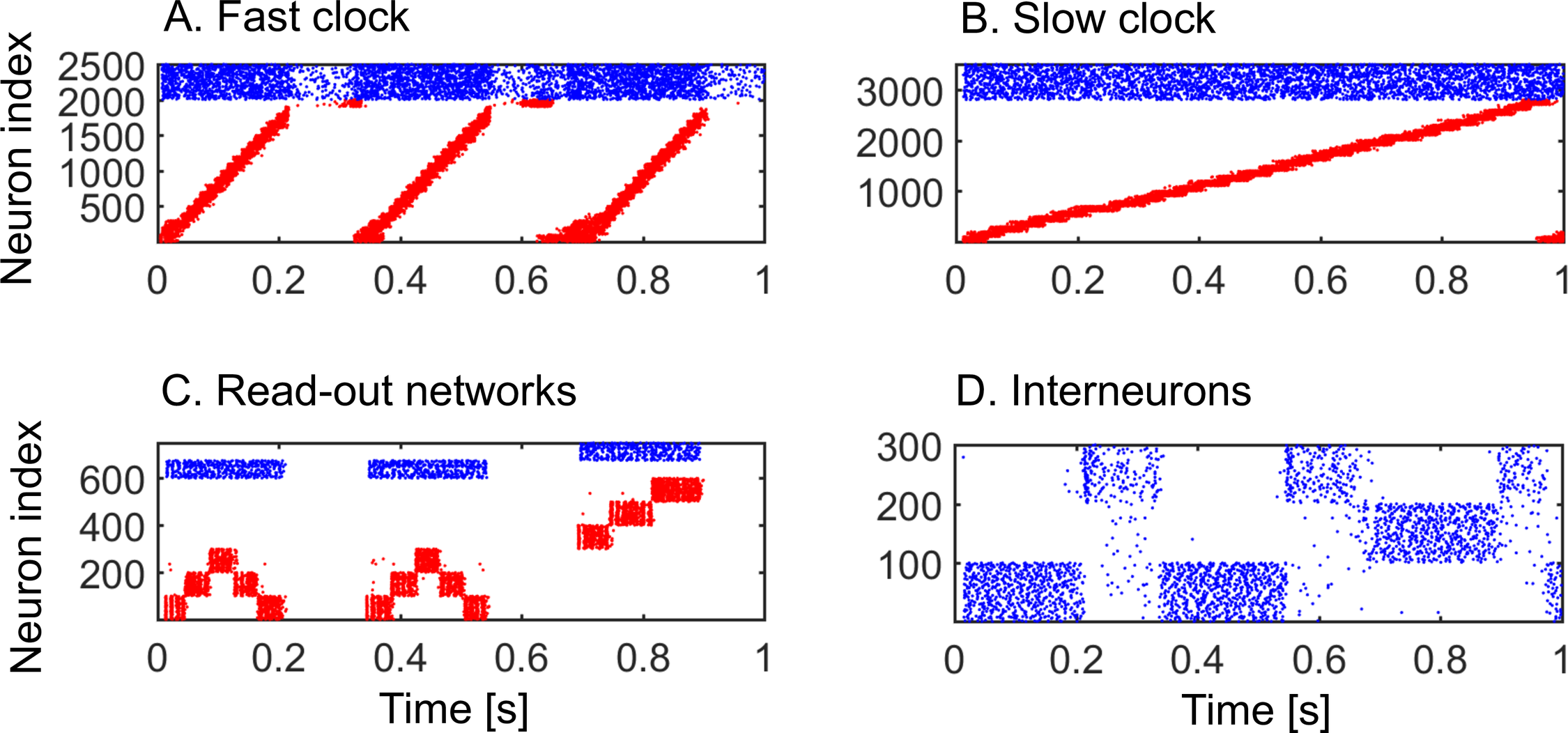

Supplement: S1 Fig — A. The first cluster of the fast clock receives a high input current at the start of each motif presentation. B. The first cluster of the slow clock receives a high input current at the beginning of the sequence presentation. C. The high input current forces spiking in the read-out neurons. D. The read-out neurons activate the interneurons. (TIF) [file pcbi.1008866.s002.tif]

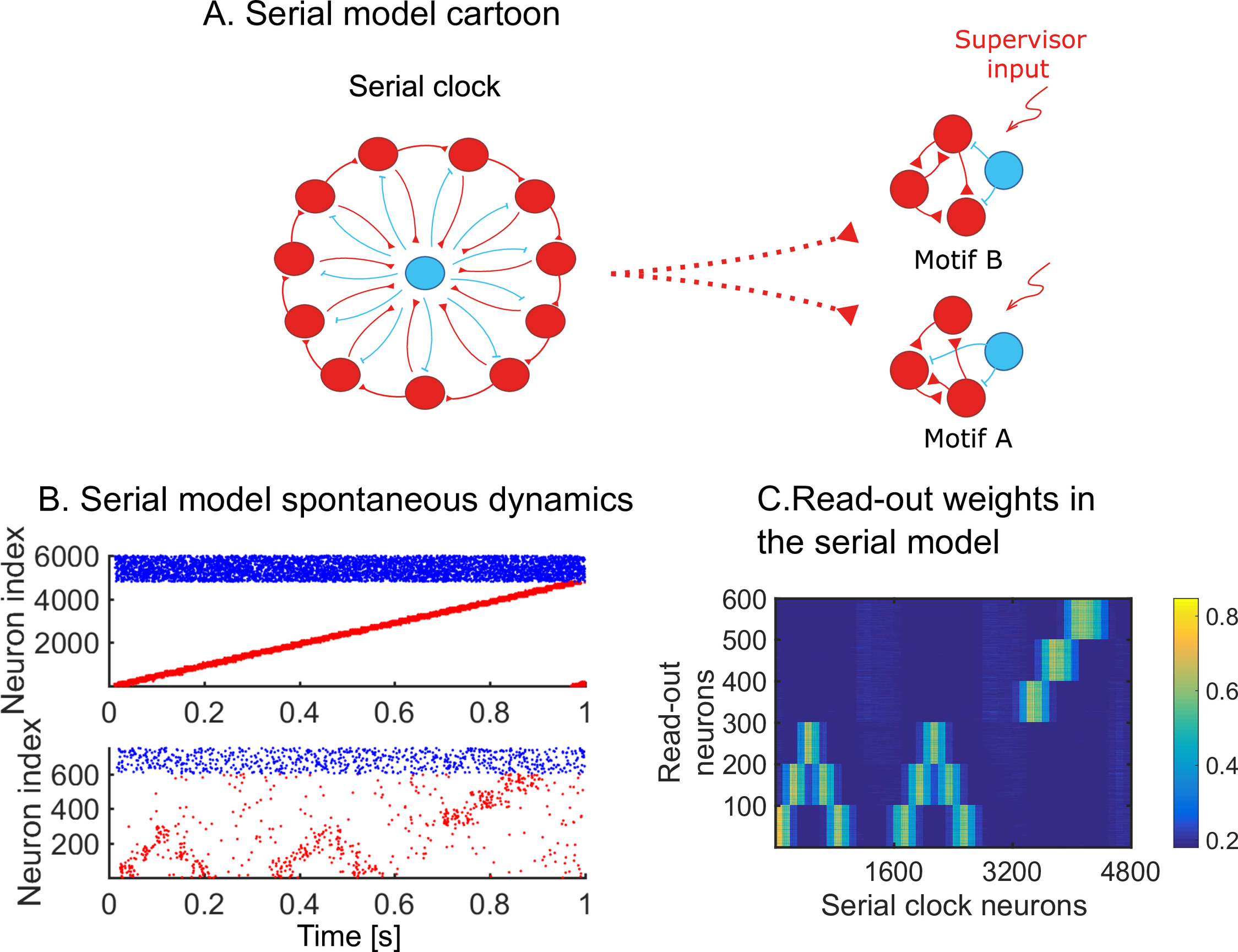

Supplement: S2 Fig — A. A single recurrent network clock (left) produces sequential dynamics and drives the dynamics in the read-out networks (right). The weights from the serial clock to the read-out network are plastic. B. We learn target sequence AAB. Spontaneous dynamics is simulated after 90 target sequence presentations. C. The read-out weights after learning. Both motif and syntax information are stored in the same weights. (TIF) [file pcbi.1008866.s003.tif]

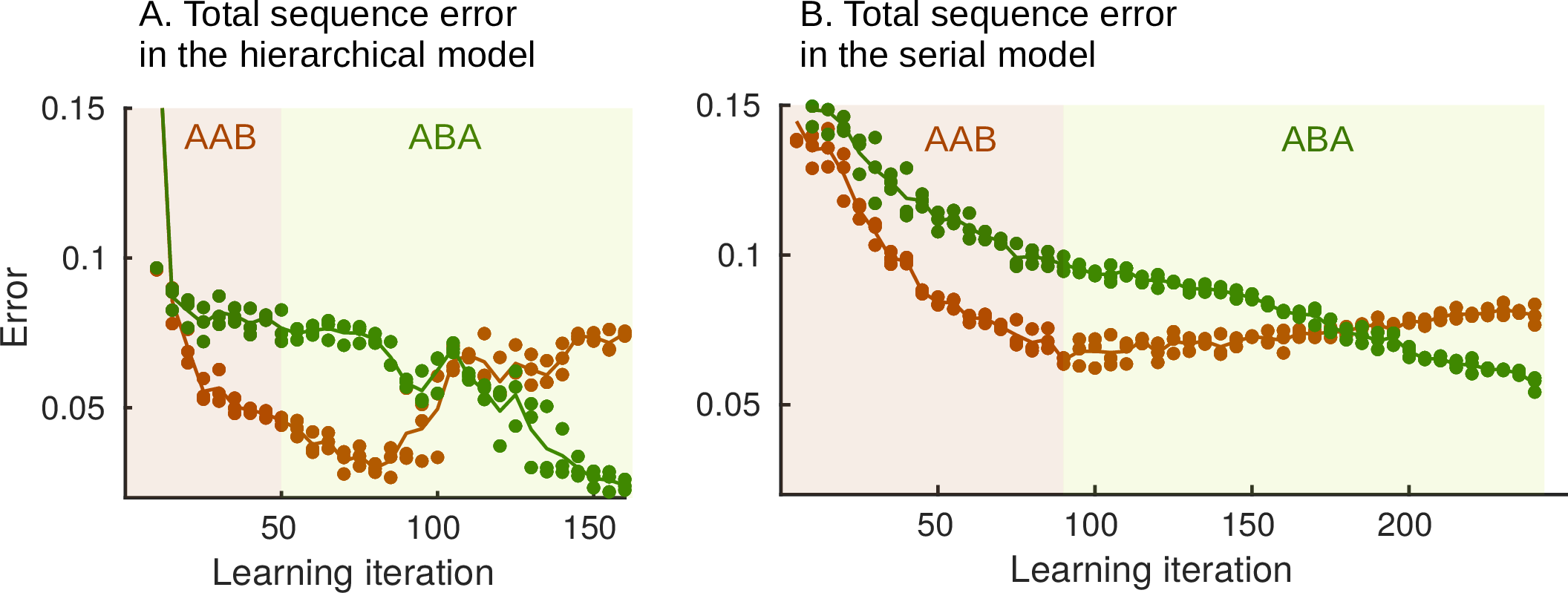

Supplement: S3 Fig — Spontaneous dynamics is simulated every fifth training iteration and compared with target sequence AAB (brown line) and target sequence ABA (dark green line) to compute the total sequence error. A. Total sequence error for the hierarchical model. Note how the total sequence error (which is the combination of within-motif error and syntax error) relative to AAB decreases for about 30 iterations after target ABA is presented for the first time due to the continued improvement in the within-motif dynamics. After this, there is a marked increase in the syntax error and the total error relative to AAB. B. Total sequence error for the serial model. The lack of hierarchy in the serial model implies that both the within-motif dynamics and motif ordering has to be relearned. This leads to a more gradual and slower relearning (note the longer x-axis). (TIF) [file pcbi.1008866.s004.tif]

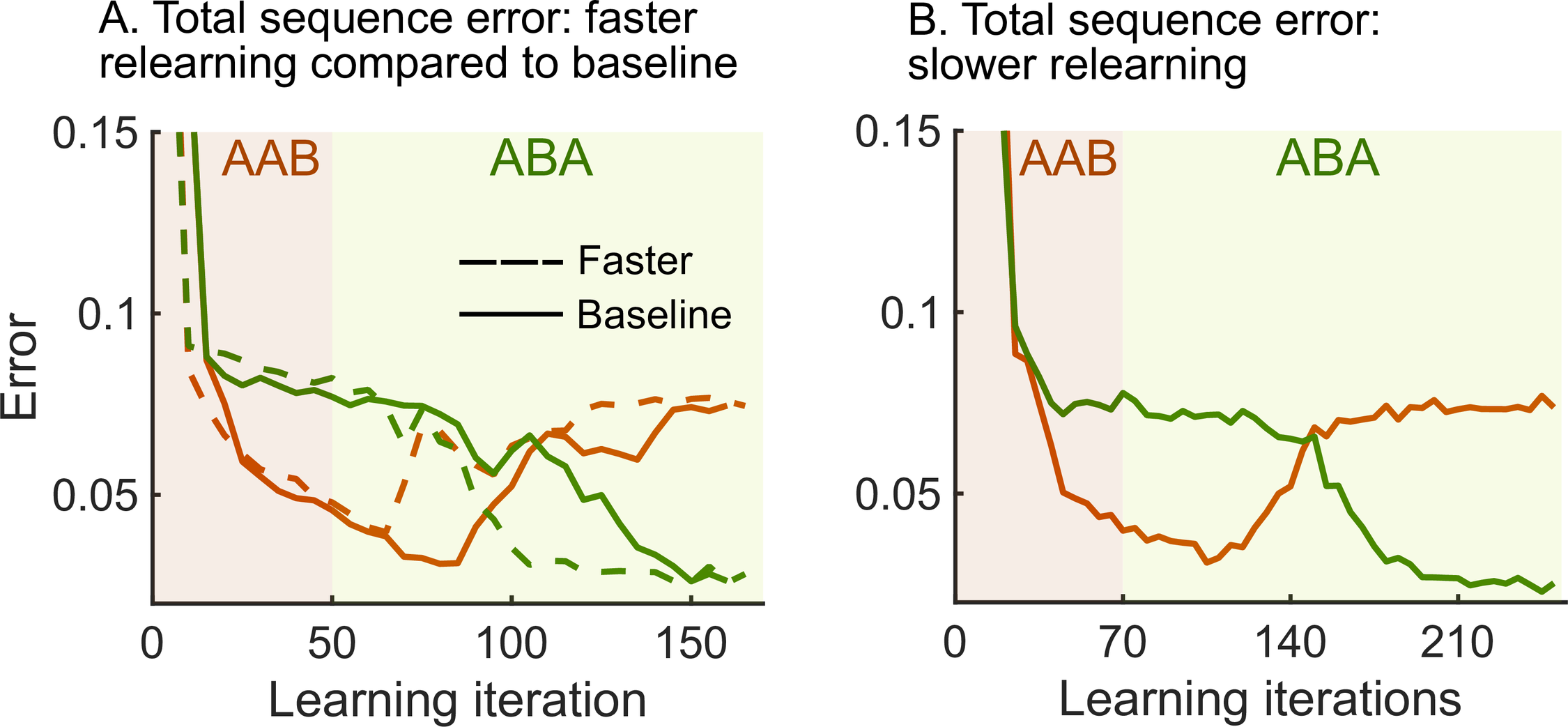

Supplement: S4 Fig — Spontaneous dynamics is simulated every fifth training iteration and compared with target sequence AAB (brown line) and target sequence ABA (dark green line) to compute the total sequence error. The lines shows the average of 5 simulations. A. The solid line shows the same total error as in S3(A) Fig (the baseline). The dashed line shows the total error, when learning faster. The right hand side of Eq 8 is multiplied by a factor 2. B. The total error when learning slower. The right hand side of Eq 8 is divided by a factor 2. More iterations are shown because the model needs more time to learn the sequences. (TIF) [file pcbi.1008866.s005.tif]

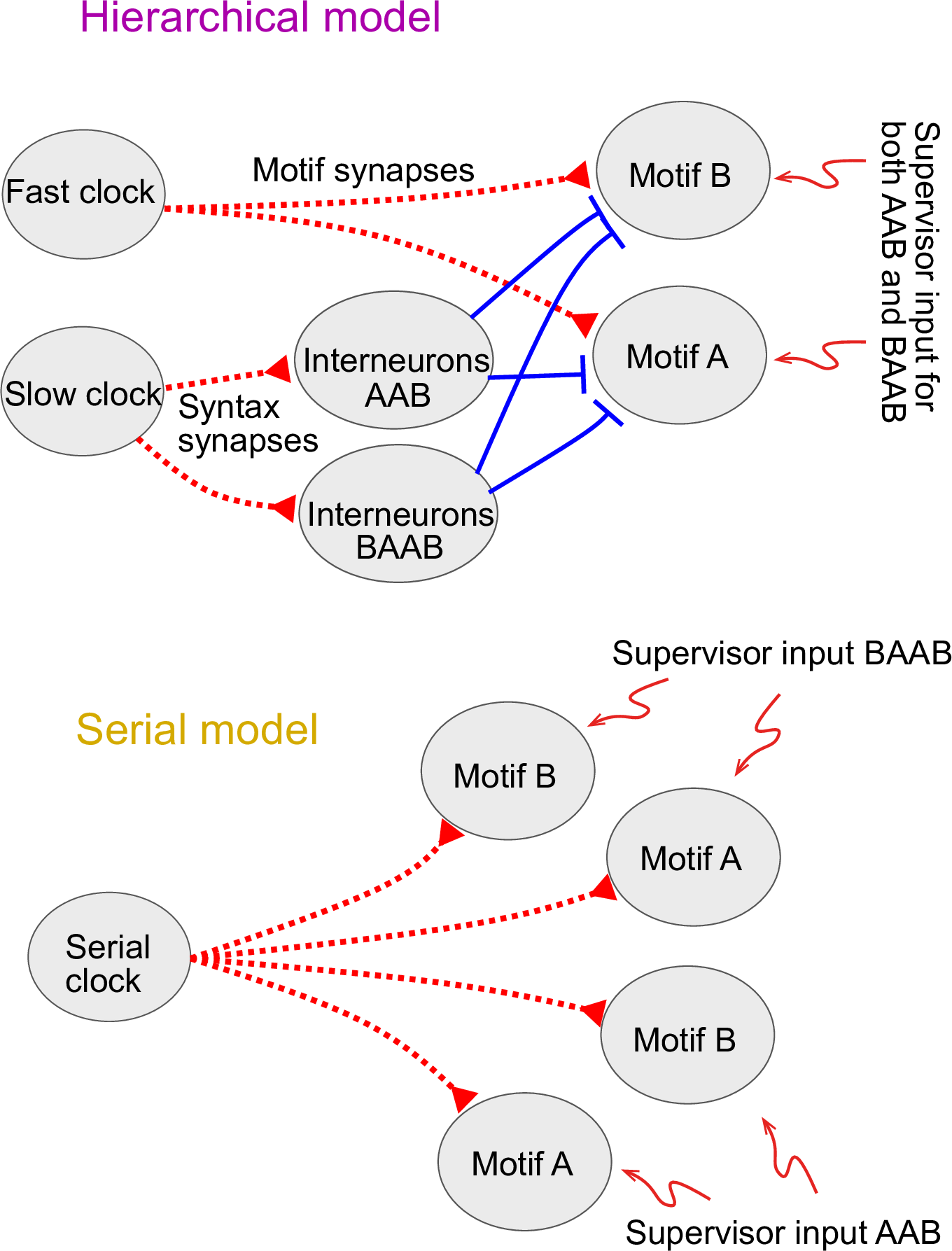

Supplement: S5 Fig — The hierarchical model requires an additional interneuron network. An external current is assumed to inhibit the interneurons for sequence BAAB when sequence AAB is presented and vice versa. The serial model duplicates the entire read-out network. Here also, an external current is assumed to inhibit the read-out networks for sequence BAAB when sequence AAB is presented and vice versa. (TIF) [file pcbi.1008866.s006.tif]

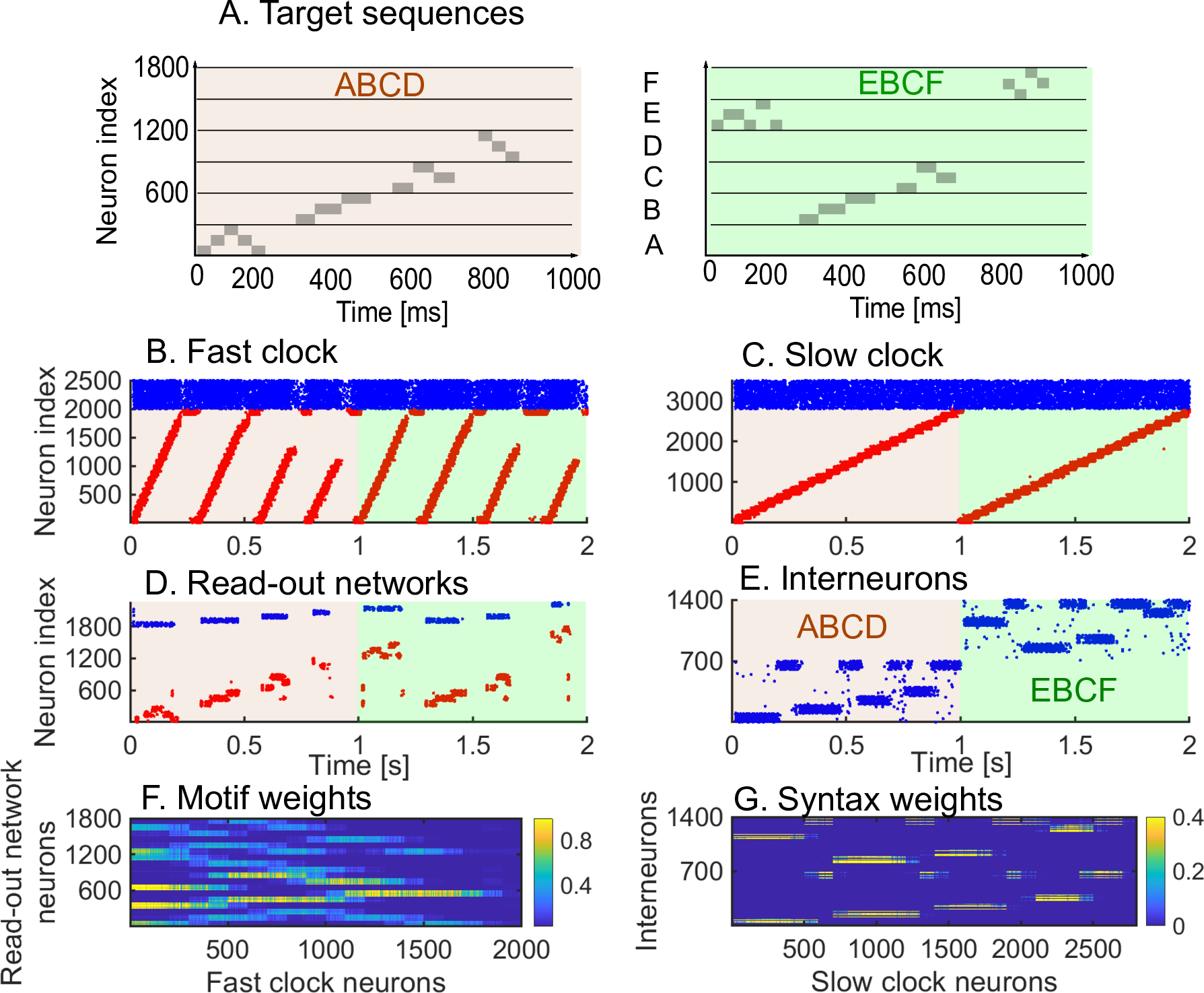

Supplement: S6 Fig — A. The two target sequences. Individual motifs have durations between 100 and 200 ms. Inter-motif intervals range from 50 to 150 ms. B-E. Red dots: excitatory neurons; blue dots: inhibitory neurons. Brown shaded area: sequence ABCD is played by inhibiting the interneurons related to the second sequence; light green shaded area: sequence EBCF is played by inhibiting the interneurons related to the first sequence. B. Spike raster of the fast clock. C. Spike raster of the slow clock. D. Spike raster of the six read-out networks. E. Spike raster of the interneurons. An external attentional inhibitory current selects which sequence is played. F. The motif weights encode the six motifs. Note that motifs B and C are learned more as they occur in both sequences. G. The syntax weights encode the two sequences. All motif and syntax synapses are plastic at all times during the sequence presentations (see S1 Text for Method details). (TIF) [file pcbi.1008866.s007.tif]

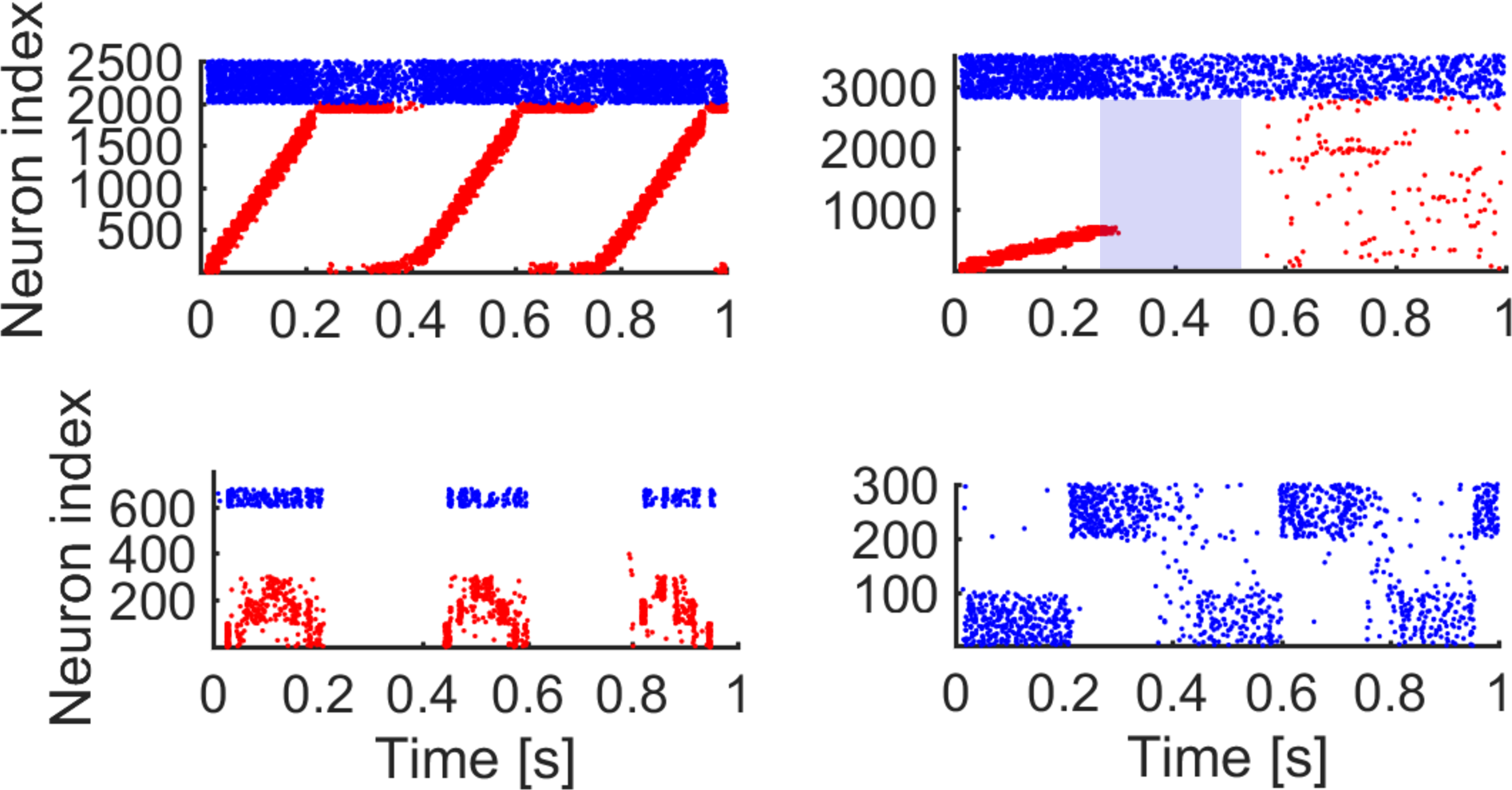

Supplement: S7 Fig — Blue shade indicates the perturbation time, all excitatory neurons receive no external input for 250 ms. The sequential dynamics in the slow clock breaks down (top right) but random activity in the interneurons (bottom right) leads to sequences in the fast clock (top left), which in turn leads to motif replays (bottom left). (TIF) [file pcbi.1008866.s008.tif]

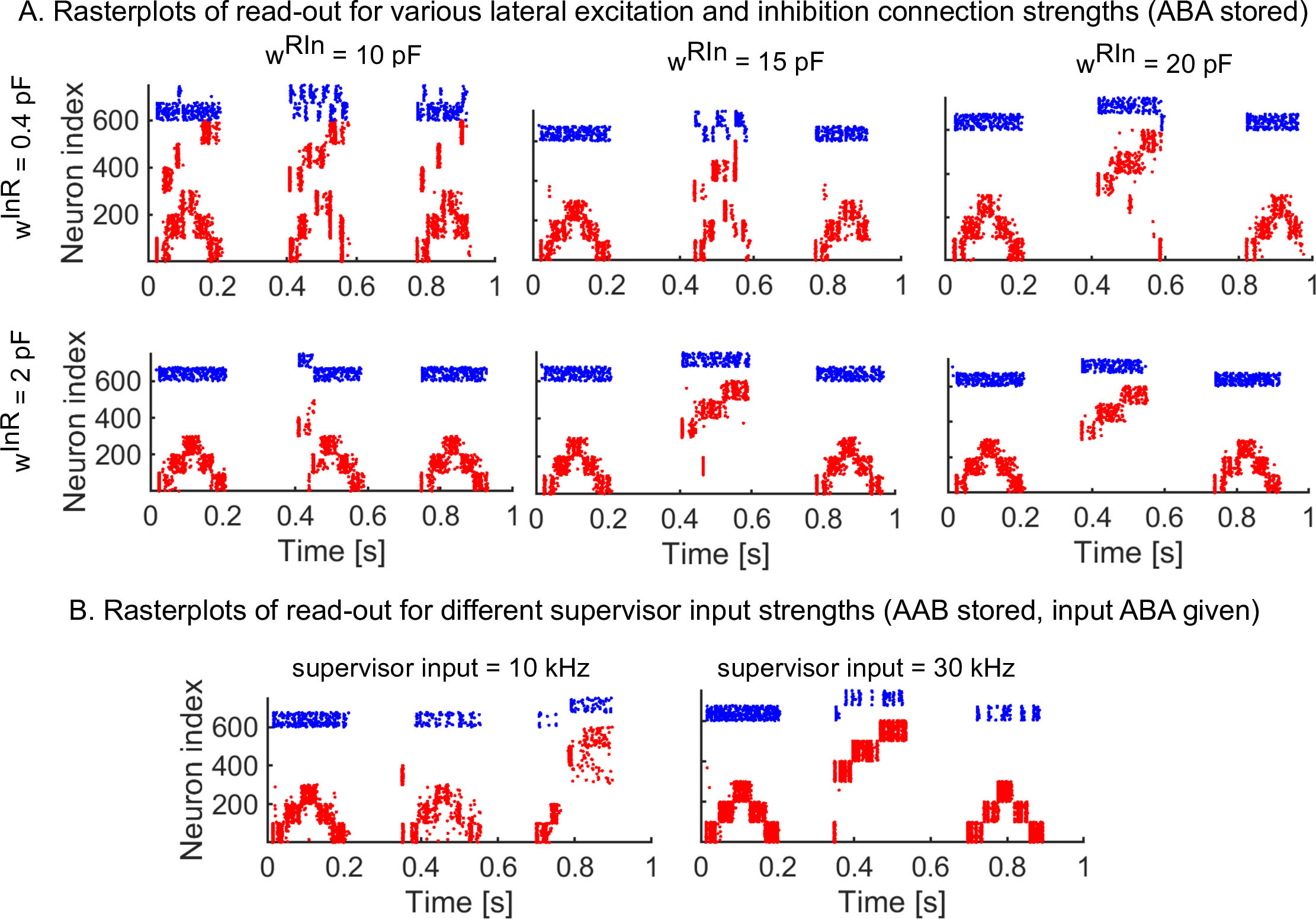

Supplement: S8 Fig — A. Spontaneous dynamics is simulated for a range of parameters, for a model that has learned sequence ABA. The potentiated motif synapses have values between 0.7 pF and 1 pF. Raster plots of the read-out network is shown. The lateral inhibition wRIn and the lateral excitation wInR are varied. When the lateral inhibition is too weak, the motifs occur at the same time (top left panel). When the lateral inhibition is sufficiently strong, the motifs are replayed well (bottom right panel). B. A supervisor gives input ABA to the read-out network, for a model that has stored sequence AAB. When the supervisor input is too low (left panel), the stored sequence dominates the dynamics in the read-out network and there will be no relearning. When the supervisor input is sufficiently high (right panel), the stored sequence is overwritten by the supervisor input and there will be relearning. (TIF) [file pcbi.1008866.s009.tif]

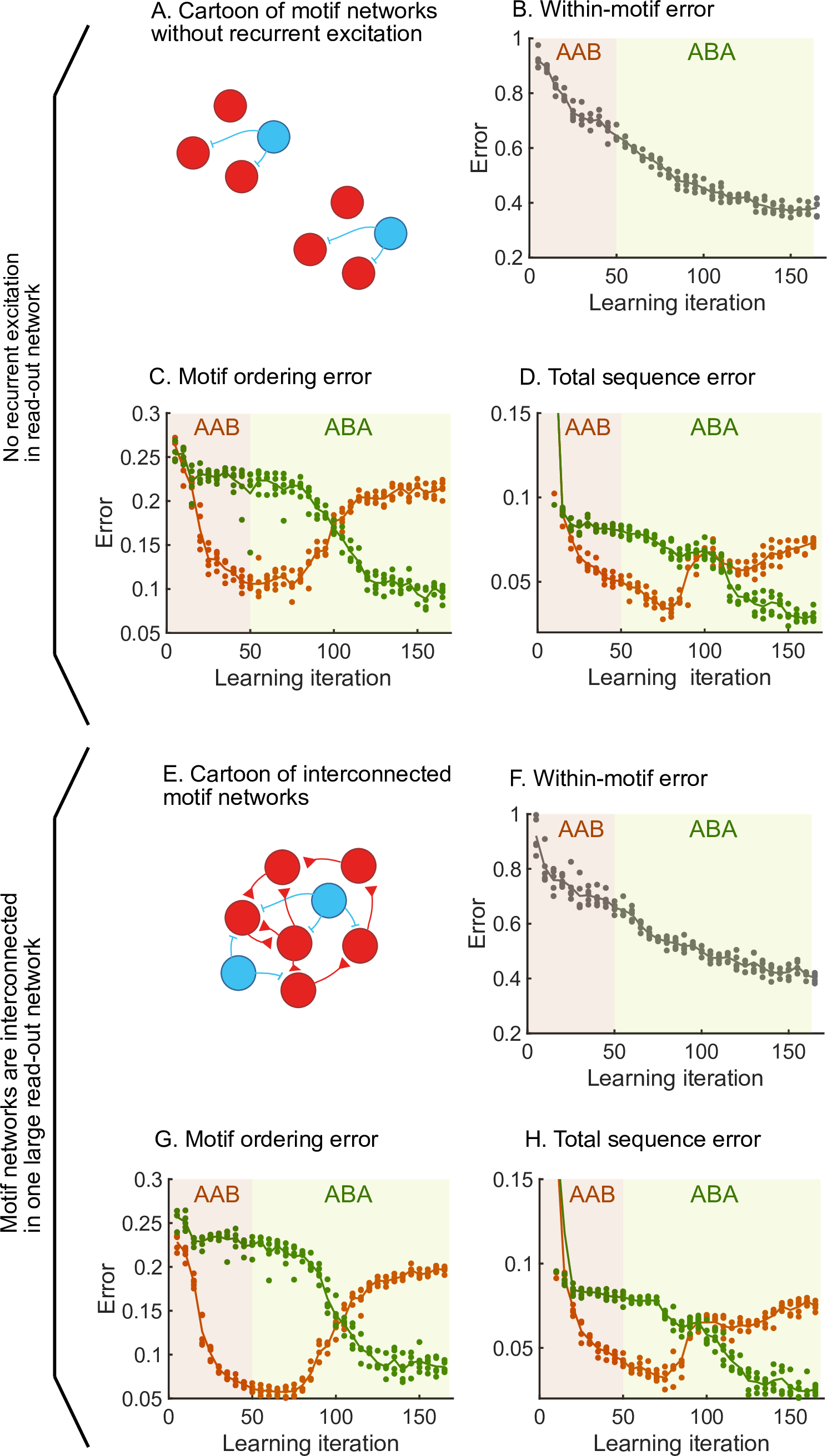

Supplement: S9 Fig — The read-out network in the main text consists of two separate networks, which are not interconnected. A. Cartoon of read-out network without recurrent excitatory connections. B-D: The learning curves when the recurrent connections in the two separate motif networks are zero. The same relearning protocol as in Fig 3 and S4 Fig is used. E. Cartoon of read-out network when the two motif networks are combined and interconnected into one network. F-H: The learning curves when the two motif networks are combined and interconnected into one network. In this case, the same connections as listed in Table 2 are used but multiplied by 1/2, and NRE=600, NRI=150. The sparsity of the connections remains p = 0.2. The same relearning protocol as in Fig 3 and S4 Fig is used. (TIF) [file pcbi.1008866.s010.tif]
