# Supplementary material for: Learning compositional sequences with multiple time scales through a hierarchical network of spiking neurons
Source: PLoS Comput Biol. 2021 Mar 25;17(3):e1008866. doi: 10.1371/journal.pcbi.1008866 (PMC8023498; doi:10.1371/journal.pcbi.1008866)
Supplement: Attachment — Submitted filename: Response_to_reviewers1.pdf [file pcbi.1008866.s011.pdf]

Responses to the reviewers

Amadeus Maes, Mauricio Barahona, Claudia Clopath

January 2021

Reviewer 1

This paper presents a computational model of sequence generation. The novel aspect of the model is that it focuses on hierarchical sequences—such as the sequences of syllables within a motif, and the sequence of motifs (syntax) that compose a songbirds song. The model assumes two clocks, a fast and slow clock responsible for the syllable sequence within a motif and the motif sequence, respectively. There is a readout network for each motif and an interneuron network for each overall sequence (syntax). Learning is supervised but biological, and takes place at the fast clock -> to read-out synapses and slow clock -> interneurons synapses. The problem being addressed is important and the model captures a number of interesting biological properties in a flexible manner. But the model is also require some significant assumptions and is fairly hardwired in some regards, for example, in regard to the connectivity between from the read-out to interneuron networks.

To allow the reader to better understand the limitations of the model and what has to be improved upon in future models the authors should discuss or address the shortcomings of the model and where it deviates from experimental data, including:

1. It appears that, as presented each motif (or the inter-motif interval) has to be the same duration. This would appear to imply a periodicity to the motifs which does not seem to be anecdotally consistent with many sequential behaviors, or with the birdsong data in which different motifs can have different durations.

We thank the reviewer for this important remark. Indeed, motifs of different duration are generally observed experimentally. We address this by extending the model such that it can learn motifs of different lengths and have different inter-motif intervals. The **new supplementary figure S6** shows an example simulation, where the model learns sequences $ABCD$ and $EBCF$. In that example, the six motifs have durations between 100 and 200 ms. One additional assumption is needed here: the supervisor must send a signal at the end of each motif during learning. This is explained in the text of the supporting information (Text S1). We have also made an observation about the limitations of this approach: *'We observe that the model is able to learn the two sequences, but the replay of shorter motifs D and F is less accurate.'*

2. I'm not aware of any evidence that supports the notion of two separate and independent fast and slow clocks in the biological literature. For example, I don't think there is any evidence of a fast and slow sequence/clock in the birdsong system. Do the authors consider this to be a prediction of their model?

We thank the reviewer for this comment. Indeed, we are not aware of any evidence concerning two separate clocks in the same system. Therefore, we can view our results as a prediction that sequences with different time scales, potentially in different brain regions, can cooperate to control different aspects of behaviour. We clarify this in the discussion: *'While sequential dynamics has been observed, coordination between multiple sequences on different scales, as we propose in this paper, has not been observed experimentally. We thus present this as a prediction that sequences on different scales may organize compositional behaviour in the same brain region or across different brain regions.'*

3. The specific hardwiring of the readout to interneuron connections along with the presence of essentially independent motif network seems like a potential challenge to the biological plausibility.

We thank the reviewer for making this remark. We recognize that the specific hardwiring and independent network motif appeared as a potential challenge to biological plausibility. To address this concern, we have simulated the model using varying connection strengths between the read-out and the interneurons (**new supplementary figure S9A**), and we summarize the results in the discussion: *'An important aspect of the hierarchical model is the interneuron network, which coordinates the different time scales. The specificity of the hardwired lateral connectivity to and from the interneuron network is a limitation of the model, but does not require extensive fine tuning, as seen in Figure S9A.'*

Additionally, we have shown that the motif networks need not be independent. Indeed, the motif networks can be joined in one large read-out network, as long as the supervisor input and interneuron input are still specific. We show this in the **new supplementary figure S8**. We have added text in Methods referring to this figure: *'The results are not sensitive to the exact configuration of the read-out networks (see Figure S8)'*.

Clarify whether the Inh units in the read-out networks receive external input, and plastic synapses from the fast clock.

The inhibitory neurons in the read-out networks receive only random external input, and no plastic synapses from the fast clock. We have now clarified this in the methods: *'The excitatory neurons in the read-out networks receive excitatory input from the plastic motif synapses coming from the fast clock. All read-out neurons receive inhibitory input from the interneurons.'*

The model will be easier to understand if in Figure 1 the projections from Motif A and Motif B, and Slow clock are shown to project exclusively and specifically to the appropriate population of units in the interneuron network. Otherwise it is difficult to understand how the supervised learning governs plasticity in the interneuron network.

We thank the reviewer for this comment. We agree that the suggested changes improve Figure 1, and we have implemented them.

In Figure 2 the authors should also show the weight matrices of the nonplastic weights from the read-out to interneuron networks, and vice-versa, as well as the interneuron to fast clock. This will greatly help the reader understand how the model works.

Thanks for the suggestion. We have added the weight matrices to figure 2, and updated the caption: *'H. Non-plastic inhibitory weights from the interneuron network to the read-out network and fast clock. I. Non-plastic excitatory weights from the read-out network and fast clock to the interneuron network.'*

Clarify what the multiple dots in each iteration in Figure 3 represent.

The three dots represent three errors measured from three independent spontaneous reactivations. We have changed the caption of figure 3 to clarify that: *'Brown dots: spontaneous dynamics is simulated* 3 *times, and the error with respect to the target sequence* AAB *is measured; dark green dots: spontaneous dynamics is simulated* 3 *times, and the error with respect to the target sequence* ABA *is measured.'*

Reviewer 2

In their manuscript the authors address the interesting problem of storing sequences (e.g. ABA) that are made up two motifs (A and B), where each motif is again a sequence (e.g. 12321 or 456). A sequence (called syntax) unfolds on a timescale of a second and each motif lasts up to a couple hundred milliseconds. Building on their recent work (Maes, Barahona, Clopath, 2020, Plos Comp. Biol.), Maes et al introduce a spiking neural network structure consisting of four interconnected components: a slow clock, a fast clock, interneuron network, and read out networks. With this network structure they learn motifs in feedforward weights from the fast clock to read out networks, with each read out network representing one motif. Feedforward weights from the slow clock to the interneuron network switch between predefined interneuron clusters. These clusters control which read out network may become active, suppressing all others. They show that this network structure learns the motifs and syntax synapses independently. Further, compared to a single network (serial model) their network structure is more resistant to perturbations. New combinations of the same motifs can be achieved without adding more readout neurons; however, this requires recruiting copies of the inhibitory network. They also find that the pattern of variability differs when compared to a single network as there is less within motif variability however more variability in the timing of motif switching, due to variability in the period of the slow clock. Overall this is a nice extension of their recent paper (Maes et al, 2020, Plos Comp. Biol.) to now include sequences of sequences on multiple timescales. Previously they used one clock network to control read outs. Switching between read out networks was done manually. The novel aspect here is that a second slower clock is added to control interneuron groups which can switch between read out networks, forming a slow sequence (syntax) of faster subsequences (motif). There remain however concerns about the clarity of presentation, robustness of the architecture, and some of the conclusions drawn, as listed in the following.

Major comments:

- It seems that the model requires specific connectivity and balance between the different weights to achieve the desired behavior. For example, there are specific feedback connections from each motif to one interneuron cluster. Activation of this interneuron cluster recruits lateral inhibition onto all competing motifs. This inhibition must be strong enough to counteract all feedforward excitation from the fast clock to the read out, in the manuscript these inhibitory weights are two orders of magnitude stronger (0.3 pF vs. 50 pF, Table 5). Thus, at least some discussion of the constraints in connectivity, weights, and activity should be added (e.g. ratio of inh/exc weights allowed? How sparse can activity in the motif be before feedback to its interneuron cluster does not recruit sufficient lateral inhibition?). In addition, limitations as well as experimental predictions stemming from these constraints might be helpful to discuss.

We thank the reviewer for raising these points. Indeed, the specific connectivity is an important element of the model. To understand the limitations of the connectivity, we have simulated the model with a range of parameters (**new supplementary figure S9A**). We add to the discussion: *'An important aspect of the hierarchical model is the interneuron network, which coordinates the different time scales. The specificity of the hardwired lateral connectivity to and from the interneuron network is an important limitation of the model, but does not require extensive fine tuning, as seen in Figure S9A.'* Additionally, we clarify the role of the activity in the motifs during learning (**new supplementary figure S9B**). A weak supervisor input does not provide enough activity in the motif network to overcome the dynamics generated by the model itself.

- While the idea is compelling, the results section on capacity of the model, the shown results do not sufficiently support the claim. Given that you are talking about how many neurons and synapses need to be added to store a new sequence, it may be more appropriate to discuss and quantify the resources required instead of capacity.

We agree with the reviewer's comment. To quantify further the resources, we have added the following text in the results section:
'We define capacity here as the number of neurons and synapses needed to store a number of sequences N_S. [...] In the case where we have a number of sequences N_S consisting of two motifs (and using network parameters as detailed in the Methods), we have the following capacities. For the serial model, we have 6000 neurons in the serial clock (of which 4800 are excitatory), and 750 neurons in each read-out network (of which 600 are excitatory). Then the number of neurons needed is $6000 + 750 \cdot N_S$, and the number of plastic synapses needed is $4800 \cdot N_S \cdot 600$. For the hierarchical model, on the other hand, there are 6000 neurons in the fast and slow clocks combined, 750 neurons in the read-out network, and 300 neurons in each interneuron network. Hence the number of neurons needed is $6750 + 300 \cdot N_S$, and the number of plastic synapses and non-plastic lateral connections due to the interneuron network is $2000 \cdot 600 + 2800 \cdot 300 \cdot N_S + 750 \cdot 300 \cdot N_S + 600 \cdot 300 \cdot N_S + 2000 \cdot 100 \cdot N_S + 200 \cdot 100 \cdot N_S = (2000 + 2441.67 \cdot N_S) \cdot 600$. For two sequences ($N_S = 2$), we then have 7500 neurons and 5,760,000 synapses for the serial model, whereas the hierarchical model requires 7350 neurons and 4,130,000 synapses—a significant reduction in resources. Even when $N_S = 1$, the hierarchical model uses more neurons than the serial model, but still fewer synapses. In general, the hierarchical model scales substantially more favourably as N_s is increased.' We also simulate a model which learns two sequences consisting of in total six motifs. Here too, we see a reduction in the number of resources: *'A serial model would need 10,500 neurons and 17,280,000 synapses. The hierarchical model uses instead 9650 neurons and 13,630,000 synapses, again a significant reduction in resources.'*

- If understood correctly, if A is repeated at different positions in a sequence (e.g. ABA), or is in different sequences (e.g. AAB vs. BAAB), different representations are required for each instance of A. While the motif for A is preserved, different instances of A are stored in the corresponding weights from slow clock clusters to the inhibitory networks. If this is correct, slow clock clusters can be reused for each instance of A, however different sets of (slow clock to inhibitory network) synapses are required to store multiple representations of A. This strategy seems to save on neurons at the expense of requiring more synapses. Please discuss.

It is indeed true that, in the example given by the reviewer, each instance of A requires a different representation. As discussed in the comment above, When looking at the number of neurons and synapses needed for the serial and hierarchical model, we see a significant decline in the number of synapses ($\sim 25\%$) and a moderate reduction in the number of neurons ($\sim 10\%$) needed. For a single sequence stored (i.e., $N_s = 1$ in the comment above), the number of neurons used in the hierarchical model is higher than in the serial model, yet the number of synapses needed is still lower.

- I agree with the authors when they write "Non-Markovian sequences are generally hard to learn, because they require a memory about past dynamics". As I understand it, in this work, for a repeating motif in a sequence (e.g. ABA), two different clusters in the slow clock potentiate their projections with interneuron cluster A. The slow clock is used as an index. Perhaps this is a matter of interpretation, but it seems that this bypasses the need for memory about past dynamics. Instead the order of the sequence elements is stored via the slow clock cluster order and is executed through feedforward weights to the interneuron groups, making

the problem Markovian. I would be interested to hear the authors' interpretation of this.

We thank the reviewer for this very insightful comment. This is indeed correct. The sequences in the fast and slow clock provide time indices to bypass the non-Markovianity on both the fast and slow time scales. We can then indeed say that, as remarked by the reviewer, the problem has been made Markovian when including the sequences. We have now added this insight in the text as follows: *'The sequential dynamics in the fast and slow clock provide a mechanism to overcome this challenge: by providing time indices the non-Markovian sequence is essentially transformed into a Markovian sequence.'*

- Related to the previous comment: can your system learn and recall overlapping sequences such as ABCD vs. EBCF?

To address this comment, we simulated a model where the sequences ABCD and EBCF are both learned. The **new supplementary figure S6** summarizes the results of this simulation. Motifs B and C occur in both sequences and are as such better learned. Two different interneuron networks are needed here to switch between the two different sequences, such as in figure 6, but overall, the model is able to learn overlapping sequences.

Minor comments:

- Related to point 1, Figure 1 could better reflect the specific connectivity required. For instance, it would be helpful to label the interneurons in the interneuron network. In this case, one belongs to motif A, one to motif B, and one to the fast clock. It would also be helpful to show that the connectivity is predetermined in a very specific way.

We thank the reviewer for this remark. We have changed figure 1 adding labels and improving the clarity of the hardwired connectivity to and from the interneurons.

- For motif error, is each motif played out independently of the slow clock sequence order? What is exactly stimulated and how?

This is correct. We have clarified this point by adding a separate paragraph for the motif error in the methods. We write: *'The fast clock and motif networks are uncoupled from the slow clock and interneuron network. We simulate spontaneous dynamics in the fast clock and motif networks by giving an input to the first cluster of the fast clock. We end the simulation after one fast clock sequence is completed.'*

- Regarding relearning syntax sequences while reusing motifs: which constraints are there on the timescales? Is it correct that the syntax must be learned faster to avoid motifs being relearned?

We thank the reviewer for this interesting question. There are no constraints on the timescales when relearning the syntax: because the motif pathway is independent of the syntax pathway, the motifs will not be relearned. The **new supplementary figure S4** shows a simulation where the learning rate of the syntax synapses is halved: the sequence is relearned but slower. We mention in the result section: *'The speed by which an old syntax is unlearned, and a new syntax is learned, depends on the learning rate of the syntax plasticity (Fig S4).'*

- Based on Figure 3B, it looks like learning is faster than relearning. Is it possible to speed

up the relearning? Does this depend on the timescale of the depotentiation term?

This is a good observation: Learning is indeed faster than relearning, because learning starts from an "empty" model since there is no sequence stored at first. Relearning is slower because it has to both "unlearn" the previously stored sequence and learn a new sequence. In the **new supplementary figure S4**, we have simulated the model with a faster learning rate at the syntax synapses to show that it is possible to speed up relearning. Increasing both the potentiation and depotentiation terms is important here, to both learn the new sequence and unlearn the old sequence. We summarize this in the results as: *'The speed by which an old syntax is unlearned, and a new syntax is learned, depends on the learning rate of the syntax plasticity (Fig S4)'*.

- For movement control, the variation of speed is an essential part. Please discuss, if and how you could control the recall speed in the proposed system.

We thank the reviewer for this important remark. It is true that the variation of speed is essential for movement control. To put our model in context, we have added the following text in the discussion: *'An important aspect of sequential behaviour is the ability to vary the speed of execution. In the serial model, the speed can easily be controlled by playing the serial clock faster or slower. In the hierarchical model, this is not as straightforward. One possibility is that the fast and slow clock coordinate the increase or decrease in speed. A different possibility could be that the speed is controlled in a network downstream of the read-out network.'*

Reviewer 3

The manuscript by A. Maes et al. presents an interesting spiking neural model for the learning of dynamical spike sequences that are modular, i.e. composed of subsequences. The basic idea is that two clock-like networks, operating at slow and fast speed, govern the progress of the overall sequence and of all individual subsequences, respectively. The selection of a subsequence happens by inhibition of the other subsequences. The responsible interneurons receive input from the slow clock; their input connections are trained such that the right networks are inhibited at the right times. The connections from the fast clock to the different output neuron networks are similarly trained to generate their individual subsequences.

Remarkably, the networks can flexibly relearn the order of subsequences while the precision of individual ones still increases (Fig. 3). This is in contrast to a purely serial model with a single clock. Further, the networks can easily learn sequences where subsequences repeat. The dynamics are robust against deletion of individual subsequences and the slow clock can be implemented with a sequence generating mechanism that is generically slow as it driven by short term depression.

The manuscript is interesting, clear and well-written. I recommend its publication essentially as it is and would like to ask only a few minor questions:

1. In Fig. 1, might it increase clarity to explicitly display the inhibitory connections from IN-A to the network Motif B and from IN-B to the network Motif A?

We thank the reviewer for this comment. We have changed figure 1, and added these explicit connections.

2. Is the recurrent excitation in the Motif networks important?

This is an interesting question. To address this point, we have simulated the model without recurrent excitation in the motif networks. Our results show that the learning curves are very similar to the original learning curves, as can be seen in the **new supplementary figure S8** (to be compared with figure 3 and supplementary figure S3). To reflect this point, we have added a sentence to the Methods commenting on this: *'The results are not sensitive to the exact configuration of the read-out networks (see Fig S8).'*

3. What resets the fast clock to start with its first group of neurons after inhibition from IN-Silence stops? Fig. 2B seems to indicate that while IN-Silence is active, the last group of the fast network stays active and initiates the first group as soon as inhibition stops.

We were not clear in our original manuscript and we thank the reviewer for raising this point. The reviewer is correct: the last group of the fast network is crucial here. This last group restarts the fast clock as soon as the IN-Silence stops being active. We have now clarified this issue in the manuscript by adding the following text in the methods: *'The last cluster in the fast clock has a special role: it is not inhibited by the 'silent' interneurons and as such remains active during silent periods. Once the silent period is over, it restarts the fast clock by activating the first cluster.'*
